# The evidential value of dental calculus in the identification process

**DOI:** 10.1038/s41598-023-48761-7

**Published:** 2023-12-08

**Authors:** Dagmara Lisman, Joanna Drath, Grażyna Zielińska, Julia Zacharczuk, Jarosław Piątek, Thierry van de Wetering, Andrzej Ossowki

**Affiliations:** 1 Department of Forensic Genetic, Pomeranian Medical University, Szczecin, Poland; 2 Department of Clinical and Molecular Biochemistry, Pomeranian Medical University, Szczecin, Poland

**Keywords:** Molecular biology, Genetics, Genotype, Population genetics

## Abstract

DNA analysis-based identification is by far the gold standard in forensic genetics and it should be performed in every case involving human remains or unidentified bodies. Bones and teeth are the preferred source of human DNA for genetic analysis. However, there are cases where the nature of the proceedings and historical significance prevent the disruption of skeletal structure. The remains may also be heavily degraded. In such situations, forensic geneticists seek alternative sources of human DNA. Teeth calculus has proven to be a viable source of DNA for identification purposes. The aim of this study was to assess the concentration of human DNA in teeth calculus and evaluate the usefulness of teeth calculus as a DNA source in the identification process. Teeth calculus was collected from skeletons exhumed between 2021 and 2022 by the PBGOT (Polish Genetic Database of Victims of Totalitarianism) team from the former Stalag IID prisoner-of-war camp in Stargard. Genetic analyses included the determination of autosomal and Y-STR markers. The total concentration of human DNA was also evaluated in samples from teeth calculus and teeth taken from the same individuals. The pilot study included 22 skeletons with a sufficient amount of calculus for isolation (specified in the protocol). Samples were taken from the largest areas of calculus deposited on lingual surfaces of mandibular incisors. The prepared samples underwent DNA extraction. Our study demonstrated that teeth calculus is a source of human DNA for remains from the World War II period. The obtained DNA concentration allowed for the determination of STR markers. It was shown that teeth calculus contains human DNA in an amount suitable for preliminary identification analyses.

## Introduction

Teeth calculus is a mineralized dental plaque that contains the host's microbiome and pathogenic microorganisms. Unlike other body surfaces, it remains unchanged and provides a stable source of human DNA^1–3^. The exact mechanism of human DNA incorporation into the calculus structure is not fully understood. One of the theories is its passive adsorption from the gingival fluid and exfoliated epithelial cells, as well as inflammatory processes in the oral cavity^2,4,5^. It may also originate from host secretions containing natural killer (NK) cells and macrophages, whose presence is associated with inflammatory processes^6,7^. It is important to emphasize that the contribution of human DNA in teeth calculus is relatively low, and the DNA contained within it is fragmented. This may be due to the fact that neutrophils and leukocytes involved in the immune response contain approximately 15 times fewer copies of the genome^8^. Its structure consists of tightly adhered calcified plaque, which also includes the oral and respiratory tract microbiomes. The highest concentration of DNA is found in subgingival plaque, which is washed by gingival fluid containing pro-inflammatory cells^2^. Such a structure protects DNA from external factors^9,10^.

DNA profiling is currently the standard for identification. Forensic geneticists typically prefer teeth and bones, especially when dealing with remains. Teeth and bones serve as good reservoirs of human DNA. However, there are cases where it is not possible to obtain such material for genetic analysis. Remains may be highly degraded, the body may be extensively decomposed, or the nature of the case under investigation may prevent the disruption of skeletal structure. The identified remains may also have historical significance or be relics^11^. Such situations prompt forensic geneticists to search for alternative sources of genetic biological samples suitable for identification purposes.

Between 2021 and 2022, the team of the Polish Genetic Database of Totalitarian Victims (PBGOT), as part of the project "Search for Polish Army Soldiers Murdered in the Former Cemetery of Stalag II D in Stargard," funded by the Ministry of Heritage and National Culture, conducted the exhumation of a total of 170 victims of the camp. The primary objective of the project was to search for murdered Polish Army soldiers buried in the former Stalag II D cemetery and identify the exhumed remains of Polish prisoners of war who ended up in German captivity as a result of their involvement in the Polish defensive war of 1939. Forensic geneticists from the Pomeranian Medical University in Szczecin undertook an attempt to isolate human DNA from teeth calculus of the exhumed remains. Stalag II D was a German prisoner-of-war camp located in Stargard, West Pomeranian Voivodeship. It operated from September 1939 to February 1945. The people held there lived in open spaces for the first few months.

The remains were from the World War II period. The length of time spent in the ground suggested that the remains were significantly was degraded. Out of the 170 skeletons, only 22 individuals had a sufficient amount of teeth calculus on their mandibular incisors. Additionally, maxillary molars were collected as reference material. All analyses were conducted at the Department of Forensic Genetics, Pomeranian Medical University in Szczecin. As a team involved in the project of the Polish Genetic Database of Totalitarian Victims (PBGOT), we have worked extensively with degraded material, as evidenced by numerous publications^12–18^. Our experience shows the importance of selecting appropriate laboratory procedures and biological material to achieve success in identification. Recognizing the significant potential of teeth calculus, we decided to investigate whether it contains a sufficient amount of human DNA for identification purposes.

## Goals

The aim of the study was to examine the content of human DNA in dental calculus from remains dating back to the period of World War II as an alternative source of human DNA. An additional aim was to compare the concentrations obtained from dental calculus and a teeth extracted from the same skeleton.

## Material and methods

The skeletons are mostly well-preserved, in anatomical alignment. The majority of them are in a complete state (all skeletal elements). Due to environmental factors, a few bone elements have undergone erosion (ribs and vertebrae).

**Figure 1:**
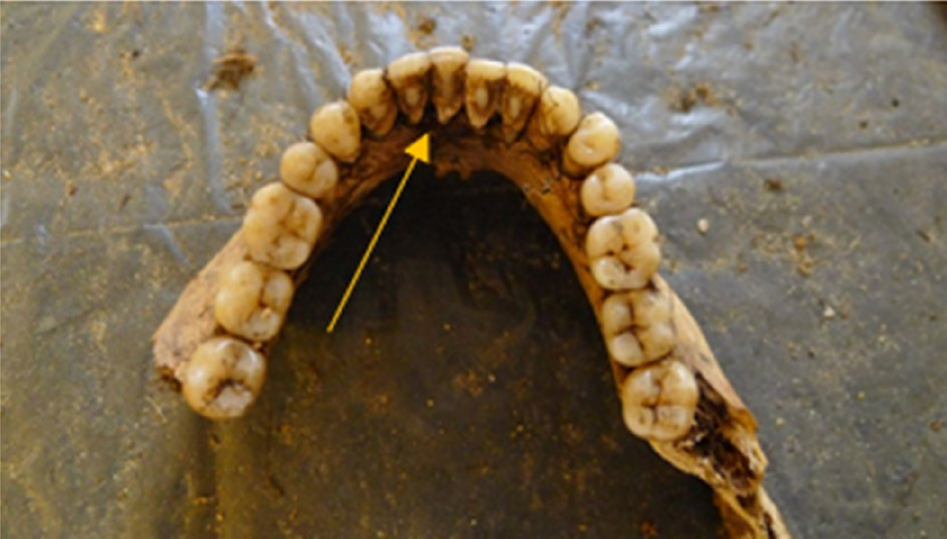
Dental calculus—mandible, view from the inside—source Department of Forensic Genetics.

**Figure 2:**
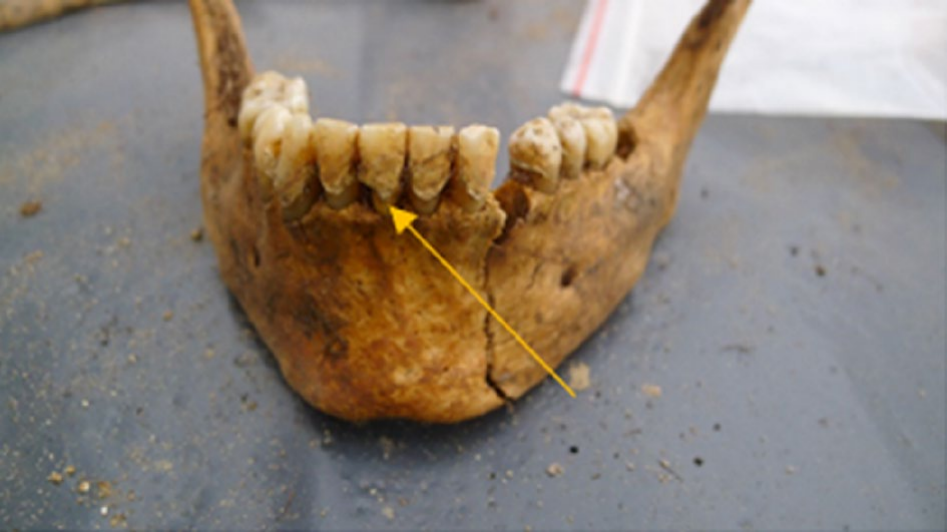
Dental calculus—mandible, view from the outside—source Department of Forensic Genetics.

The study involved 17 individuals from whom dental calculus and molar teeth from the lower jaw were collected. The collected teeth served as reference material. Dental calculus samples were collected from the thickest layer, located on the lingual surfaces of the mandibular incisors. Remains with a low dental calculus content were excluded from the study (Figs. 1 and 2).

The minimum required content for isolation was established at 10–15 mg. The equipment used for dental calculus collection included a sterile scalpel, sterile aluminum foil, and sterile Eppendorf tubes.

The exhumation was carried out at the request of the Ministry of Heritage and National Culture as part of the project "Searching for Polish Army soldiers murdered and buried in the former Stalag II D cemetery in Stargard", under which an agreement (07794) /21/FPK/DDK of July 19, 2021, was signed year). The biological material used in the work—dental calculus and teeth—comes from human remains. The research was approved by the Ministry of Heritage and National Culture. In Poland, working on material from deceased persons does not require additional consent or bioethics committees.

### Sample collection

#### Dental calculus samples

**Figure 3:**
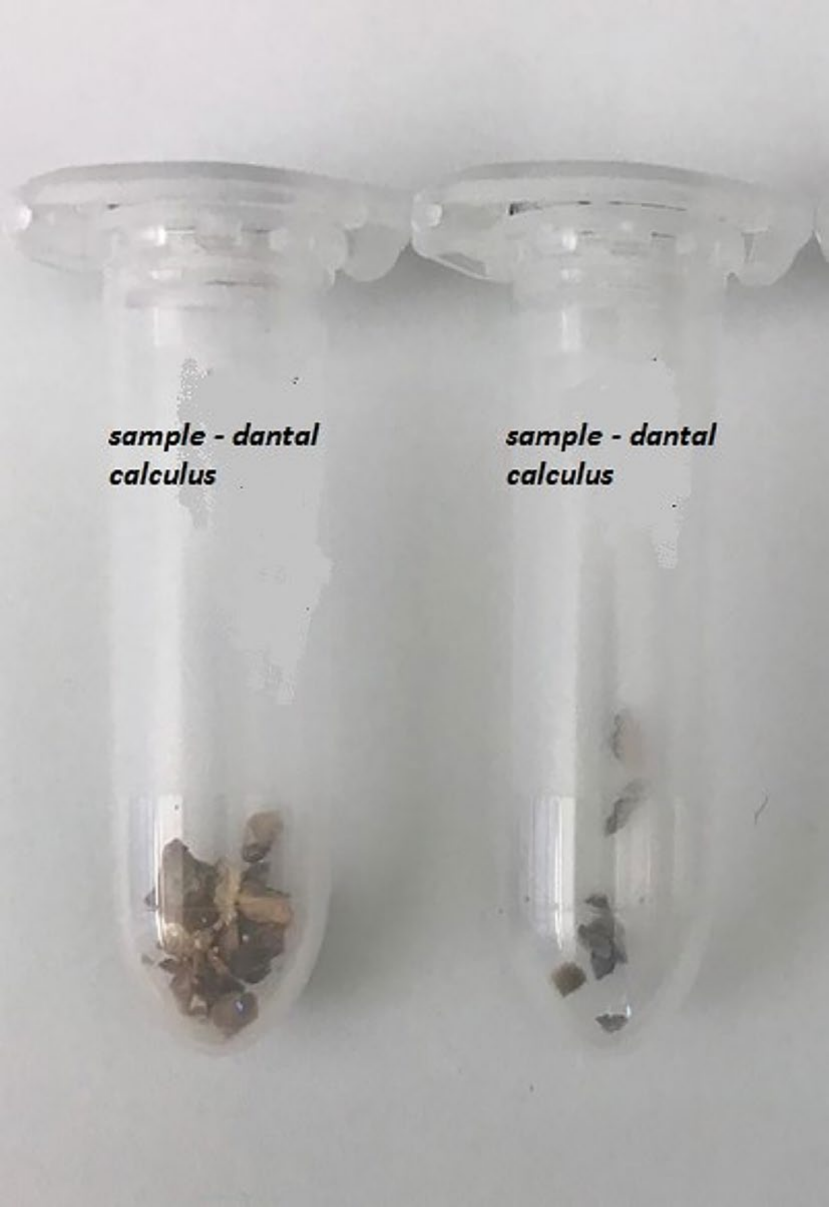
Dental calculus collected and preserved in a sterile tube- source Department of Forensic Genetics.

Using a sterile scalpel, pressure was applied to the edge of the thickest part of the dental calculus, and a separate sample of approximately 2 mm was collected. Sterile aluminium foil was placed along the teeth to collect all the components of the calculus. The collected calculus was transferred into a sterile Eppendorf tube, sealed, and labeled (Fig. 3). After collecting the entire sample of dental calculus, it was transferred onto a sterile gauze pad and exposed to UV light for 5 min to prevent microbial growth. Subsequently, the calculus samples were transferred to a new sterile Eppendorf tube, sealed, labeled, and frozen at – 20 °C until isolation.

##### Extraction of teeth

Molar teeth from the same skeleton were extracted. The teeth were collected in sterile Falcon tubes and frozen at – 80 °C until isolation.

##### DNA isolation from dental calculus

Immediately before isolation, approximately 15 mg of dental calculus was collected and placed in a new sterile tube. Then, using a sterile diamond drill at low rotation speed (5000–10,000 rmp), the calculus was ground into fine particles. The next step involved washing the particles twice with EDTA solution (0.5 M, pH 8) (Merck KGaA, Darmstadt, Germany) for one minute on a rotary mixer. The supernatant was then removed, and automated isolation was performed according to the Maxwell® FSC DNA IQ™ Casework Kit protocol (Promega), which is specifically designed for highly calcified samples. Negative controls were included for the extraction process.

All protocols were done according to manufactures instructions, without any modification.

##### DNA isolation from teeth

The preserved teeth underwent mechanical and chemical cleaning, followed by cryogenic grinding in the presence of liquid nitrogen. DNA was isolated using the Maxwell® FSC DNA IQ™ Casework Kit (Promega) with a bone powder amount of 50 mg, following the manufacturer's protocol. Negative controls were included for the extraction process. All protocols were done according to manufactures instructions, without any modification.

##### DNA concentration measurement and PCR inhibition evaluation

The Quantifiler Human DNA Quantification Kit (Applied Biosystems) and the 7500 Real-Time PCR System (Applied Biosystems) were used to evaluate the concentration of human DNA and the presence of PCR inhibitors. A CT value of ⩾ 31 was adopted as the criterion for the presence of PCR inhibitors, in accordance with the manufacturer's instructions and internal method validation.

##### STR amplification and product detection

Autosomal STR markers were amplified using the GlobalFiler™ PCR Amplification Kit (Thermo Fisher Scientific), while Y-STR markers were amplified using the Y-filer™ Plus PCR Amplification Kit (Thermo Fisher Scientific). The reactions were carried out according to the manufacturer's instructions.

Product detection was performed on a 3500 Genetic Analyzer using the GeneScan™ 600 LIZ® size standard, following the manufacturer's protocol. The results were then analyzed using GeneMapper ID-X v1.6 software.

## Results

The average DNA concentration in the teeth samples was higher than that obtained from dental calculus, but this did not translate into the quality of the obtained profiles. Obtained from dental calculus samples, the average DNA concentration was 0.03844 ng/μl lower than the average DNA concentration obtained from teeth. The total concentration of human DNA in dental calculus ranged from 0.00311 to 0.01967 ng/μl, with an average value of 0.03286 ng/μl (Table 1). While the total concentration of human DNA in the collected teeth ranged from 0.00821 μg/ng/μl ml to 0.26937 ng/μl with an average value of 0.07130 ng/μl (Table 2). In the case of sample 7/DC, it was not possible to amplify long DNA fragments or determine the average DNA concentration. A degraded profile was obtained from this sample. The degradation index in dental calculus samples ranged from 0.47344 to 3.85240 (Table 1), which was much lower compared to teeth samples, which ranged from 1.99240 to 38.15341 (Table 2). DNA degradation was determined by considering locus dropout and peak height ratio. These values were compared with the sum of peak heights at each locus, with a reference value of 100 RFU.

**Table 1:** Total human DNA concentration for dental calculus samples (DC).

Number	Sample number	Biological sex	Sample type	Amount of DNA present (ng/μl)	ID
1	4	Male	Dental calculus	0.01909	1.92022
2	6	Male	Dental calculus	0.00777	0.96091
3	7	Male	Dental calculus	0.01943	N/A*
4	11	Male	Dental calculus	0.00913	3.00144
5	16	Male	Dental calculus	0.00524	3.85240
6	23	Male	Dental calculus	0.00311	2.01058
7	25	Male	Dental calculus	0.018782	1.73430
8	27	Male	Dental calculus	0.02608	1.22179
9	30	Male	Dental calculus	0.02340	1.88926
10	32	Male	Dental calculus	0.01967	1.54357
11	33	Male	Dental calculus	0.06476	1.95332
12	47	Male	Dental calculus	0.06382	0.94826
13	53	Male	Dental calculus	0.08890	2.3566
14	58	Male	Dental calculus	0.07057	1.86514
15	61	Male	Dental calculus	0.01377	1.31236
16	63	Male	Dental calculus	0.05845	0.47344
17	91	Male	Dental calculus	0.05456	1.72763
			Mean concentration	0.03286	1.6924

*Undefined concentration, *DI* DNA degradation index.

**Table 2:** Total human DNA concentration for teeth samples (T).

Number	Sample number	Biological Sex	Sample type	Amount of DNA present (ng/μg)	DI
1	4	Male	Teeth	0.11940	38.15341
2	6	Male	Teeth	0.21033	21.67173
3	7	Male	Teeth	0.04952	6.33898
4	11	Male	Teeth	0.12491	7.22350
5	16	Male	Teeth	0.23112	3.85240
6	23	Male	Teeth	0.00431	13.93101
7	25	Male	Teeth	0.03661	3.02658
8	27	Male	Teeth	0.07304	2.79218
9	30	Male	Teeth	0.26937	2.07854
10	32	Male	Teeth	0.04597	7.99526
11	33	Male	Teeth	0.21196	23.54700
12	47	Male	Teeth	0.02277	3.51971
13	53	Male	Teeth	0.0344	2.7421
14	58	Male	Teeth	0.12829	3.56353
15	61	Male	Teeth	0.26154	3.82658
16	63	Male	Teeth	0.22131	1.25522
17	91	Male	Teeth	0.05947	1.12616
			Mean concentration	0.07130	8.6261

*DI* DNA degradation index.

In the Global Filer individual identification system (ThermoFisher), 17 male DNA profiles were obtained from dental calculus and teeth samples collected from one skeleton, representing approximately 77% of the study population (Tables 3, 4, 5, 6, 7, 8). This indicates that these samples contained genomic DNA. Fifteen profiles were obtained after analyzing the Y-STR markers, which accounted for approximately 68% of the study population (Tables 9, 10, 11, 12, 13). Consensus profiles were obtained from the teeth samples (three independent repetitions). Only one isolation was performed for the dental calculus samples due to the limited amount of material collected. All amplified STR markers (Tables 3, 4, 5, 6, 7, 8) and Y-STR markers (Tables 9, 10, 11, 12, 13) showed reproducibility in both the teeth (T) and dental calculus (DC) samples collected from the same skeleton.

**Table 3:** Summary of results in the Global Filer individual identification system—teeth (T) and dental calculus (DC) samples.

Sample	53/DC	53/T	33/DC	33/T	47/DC	47/T
D3S1358	17,18	17,18	16	15,16	16	16
vWA	17	16,17	16	16,18	16,17	16,17
D16S539	9	9,11	10	10,12	12,13	12
CSF1PO	–	10,11	10	–	10	–
TPOX	–	8	–	–	8	–
INS/DEL	2	2	2	2	2	2
AMELO	XY	XY	XY	XY	XY	XY
D8S1179	10,14	10,14	13	13	11,14	11,14
D21S11	30,31	30,31	32	30,32	29,33.2	29,33.2
D18S51	13	13,16	–	15	13,15	–
DYS391	–	11	–	–	11	–
D2S441	11,14	11,14	10,11	10,11	10,14	10,14
D19S433	14	11,14	15	15	14	14
TH01	6,8	6,8	9.3	7,9.3	6	6
FGA	24,24	24,25	22,26	22	22,24	24
D22S1045	15,16	15,16	15,17	15,17	11,12	11,12
D5S818	10	10,12	11,13	13	11,12	11,12
D13S317	11	8,11	–	12	12,13	13
D7S820	9,10	9,10	–	10	9,11	9
SE33	25.2	18,25.2	–	17	19	–
D10S1248	13,15	13,15	14	14	14	14
D1S1656	15.3	15,15.3	13	13,17.3	15.3,17.3	15.3,17.3
D12S391	20	15,20	18	18,20	21	–
D2S1338	25	19,25	–	18	17,25	–

*DC* dental calculus sample, *T* teeth sample, (–) no amplification obtained possible DNA degradation.

**Table 4:** Summary of results in the Global Filer individual identification system—teeth (T) and dental calculus (DC) samples—continued.

Sample	58/DC	58/T	4/DC	4/T	7/DC	7/T
D3S1358	15,18	15,18	–	16,18	15	16,17
vWA	16	16	–	16,17	–	19
D16S539	11,13	11	–	13	11	11
CSF1PO	12	–	–	10,12	12	–
TPOX	8	–	–	9	–	–
INS/DEL	2	2	2	2	–	2
AMELO	XY	XY	XY	XY	X	XY
D8S1179	13,14	12,13	12	12,14	–	12
D21S11	–	–	–	28,29	30	30
D18S51	14,15	–	29	–	–	–
DYS391	–	15	–	11	–	–
D2S441	12,14	12,14	11	11	10,14	14
D19S433	13,15	13,15	–	13	13,14	14,15.2
TH01	6,9.3	9.3	6	6,9.3	9	6,9
FGA	20,22	20,22	22	–	22	22,23
D22S1045	11,15	11,15	11,16	11,16	18	15
D5S818	11	11	11	10,11	13	12,13
D13S317	11	–	–	8,11	–	10,11
D7S820	9	–	–	10	–	10
SE33	15	30.2	26.2	–	–	31.2
D10S1248	13,14	13,15	14,18	14	14	14,16
D1S1656	12	12,13	18.3	16.3,18.3	16	14,15
D12S391	21,22	22	–	17	–	21
D2S1338	17	–	–	22	–	18

*DC* dental calculus sample, *T* teeth sample, (–) no amplification obtained possible DNA degradation.

**Table 5:** Summary of results in the Global Filer individual identification system—teeth (T) and dental calculus (DC) samples—continued.

Sample	23/DC	23/T	27/DC	27/T	6/DC	6/T
D3S1358	16	–	18	15	–	15
vWA	16	–	18	14,18	14,18	14,18
D16S539	11,13	–	–	11	11,12	12
CSF1PO	–	–	–	11	–	11
TPOX	8	–	10	–	10	8,10
INS/DEL	2	2	2	2	–	2
AMELO	XY	XY	XY	XY	XY	XY
D8S1179	12,13	12,13	13	13,15	12	12
D21S11	28	32.2	28	28	27	29
D18S51	15	–	14	14,16	–	12,19
DYS391	–	–	12	–	–	9
D2S441	14	–	10,12	10	10	10,14
D19S433	13	13	13	13,14	–	13
TH01	–	–	9.3	8,9.3	7	6
FGA	23	–	20	19.3	22.2	22.2,24
D22S1045	15,16	–	16,18	16,18	15	15
D5S818	11,12	11	11	9,11	12	12,14
D13S317	8,12	–	11	12	–	–
D7S820	9	–	–	11.2	–	–
SE33	–	–	–	26.2	–	–
D10S1248	15,17	–	13	13,17	15	14,15
D1S1656	11,16.3	–	–	12,15.3	18.3	17.3,18.3
D12S391	22	–	–	18,22	21	21
D2S1338	17	–	–	17,18	15	15,20

*DC* dental calculus sample, *T* teeth sample, (–) no amplification obtained possible DNA degradation.

**Table 6:** Summary of results in the Global Filer individual identification system—teeth (T) and dental calculus (DC) samples—continued.

Sample	25/DC	25/T	11/DC	11/T	32/DC	32/T
D3S1358	–	15,16	16	16,17	14,15	14,15
vWA	17,18	17,18	–	14,18	–	14,15
D16S539	–	11,14	–	11,13	–	12,13
CSF1PO	–	11	–	10	12	10,12
TPOX	–	–	–	9	8	8,12
INS/DEL	–	2	2	2	2	2
AMELO	–	XY	Y	XY	XY	XY
D8S1179	12,13	12,13	13	13,15	13	13,14
D21S11	–	28,30	–	29,30.2	29	29
D18S51	18	17,18	–	13	–	15,16
DYS391	–	10	–	–	–	11
D2S441	10,11	10,11	11	11,14	12,14	12,14
D19S433	14.3	15	–	14.2,15	12,13	12,13
TH01	–	–	9.3	9,9.3	8	6,8
FGA	19,20	19,20	–	23,25	22	22
D22S1045	11,14	11,14	15,16	15,16	15,17	15,17
D5S818	13	10,13	–	10,11	12	11,12
D13S317	9,11	9,11	11	11,12	9	9,12
D7S820	–	11	–	8	8	8,12
SE33	–	25.2	–	19	24.2	24.2
D10S1248	16	16	13	13	14	14,16
D1S1656	14,16	14,16	–	17.3,19.3	–	12,17.3
D12S391	–	17,22	–	18,19	–	21,23
D2S1338	20	17,20	–	17	–	17,24

*DC* dental calculus sample, *T* teeth sample, (–) no amplification obtained possible DNA degradation.

**Table 7:** Summary of results in the Global Filer individual identification system—teeth (T) and dental calculus (DC) samples – continued.

Sample	91/DC	91/T	63/DC	63/T	61/DC	61/T
D3S1358	16	16	17	17	17	14,17
vWA	16,17	16,17	14,17	14,17	14	14,16
D16S539	11	11,12	9,11	9,11	–	9
CSF1PO	11	11	10	10	–	9,11
TPOX	8	12	–	8,9	–	8
INS/DEL	2	2	2	2	2	2
AMELO	XY	XY	XY	XY	XY	XY
D8S1179	13	13	10,15	10,15	12,13	12,13
D21S11	29,3	29,30	28,30	28,30	28,32.2	28,32.2
D18S51	15	14,15	18	15,18	–	16,18
DYS391	10	10	10	10	–	11
D2S441	11,11.3	11,11.3	11,14	11,14	12	10,12
D19S433	13.2,14	13.2,14	13,13.2	13,13.2	14	14
TH01	8	8	6	6	9	9
FGA	24,25	24,25	17,22	17,22	–	20,24
D22S1045	11	11	11,15	11,15	16	16
D5S818	12	12,13	11,12	11,12	9,11	9,11
D13S317	9	8,9	9,11	9,11	–	11,13
D7S820	–	11,13	10	10	10	10
SE33	–	24.2,28.2	–	17,23.2	–	15,16.2
D10S1248	14,15	14,15	13,15	13,15	11,14	11,14
D1S1656	18.3	16,18.3	11,15	11,15	14,17.3	14,17.3
D12S391	18	18	–	18,25	17	17,18
D2S1338	–	23,25	18	18,25	25	17,25

*DC* dental calculus sample, *T* teeth sample, (–) no amplification obtained possible DNA degradation.

**Table 8:** Summary of results in the Global Filer individual identification system—teeth (T) and dental calculus (DC) samples—continued.

Sample	30/DC	30/T	16/DC	16/T	23/DC	23/T
D3S1358	16	15,16	–	15,18	16	–
vWA	–	17,18	–	18,19	16	–
D16S539	11	11,12	–	11,12	11,13	–
CSF1PO	11	10,11	11	11,12	–	–
TPOX	–	8	–	8	8	–
INS/DEL	–	2	–	2	2	2
AMELO	XY	XY	–	XY	XY	XY
D8S1179	–	10,14	–	12,13	12,13	12,13
D21S11	–	30.2,32.2	–	29,30.2	28	32.2
D18S51	–	16,17	–	12,19	15	–
DYS391	–	10	–	–	–	–
D2S441	11	11,14	11	10,11	14	–
D19S433	13,14	13,14	–	13,15	13	13
TH01	6	6,7	6,7	6,7	–	–
FGA	–	21,22	–	19,24	23	–
D22S1045	11	11,12	–	15	15,16	–
D5S818	–	11	11,12	11,12	11,12	11
D13S317	–	10,14	–	11,13	8,12	–
D7S820	8	8,10	–	10	9	–
SE33	–	19,31.2	–	19.2,30.2	–	–
D10S1248	–	13,14	–	13,15	15,17	–
D1S1656	12	12	–	19.3	11,16.3	-
D12S391	–	18,22	–	20	22	-
D2S1338	–	17,20	–	17,24	17	-

*DC* dental calculus sample, *T* teeth sample, (–) no amplification obtained possible DNA degradation.

**Table 9:** Summary of results in the Y Filer Plus system—teeth (T) and dental calculus (DC) samples.

Sample	6/T	6/DC	4/T	4/DC	7/T	7/DC
DYS576	16	–	18	–	18	–
DYS389I	14	14	13	–	12	–
DYS635	21	–	23	23	–	23
DYS389II	–	31	30	–	–	–
DYS627	–	–	–	–	–	–
DYS460	10	–	12	12	11	–
DYS458	17	17	15	–	16	–
DYS19	13	–	16	–	–	–
YGATAH4	–	12	12	–	–	–
DYS448	–	20	20	–	19	–
DYS391	9	–	–	–	–	–
DYS456	16	16	16	16	15	–
DYS390	24	24	25	25	–	–
DYS438	–	10	–	–	12	–
DYS392	–	–	11	–	–	–
DYS518	–	–	–	–	–	–
DYS570	17	17	21	–	17	–
DYS437	14	14	14	–	15	–
DYS385	17	17	–	–	14	–
DYS449	30	–	–	–	–	–
DYS393	14	14	13	–	12	–
DYS439	11	11	10	–	12	–
DYS481	25	–	–	–	21	–
DYF387S1	37	–	–	–	35	–
DYS533	11	–	–	–	–	–

*DC* dental calculus sample, *T* teeth sample, (–) no amplification obtained possible DNA degradation.

**Table 10:** Summary of results in the Y Filer Plus system—teeth (T) and dental calculus (DC) samples—continued.

Sample	11/T	11/DC	16/T	16/DC	25/T	25/DC
DYS576	20	–	19	19	17	17
DYS389I	13	–	13	–	13	13
DYS635	23	–	24	–	–	–
DYS389II	30	–	30	–	–	–
DYS627	17	–	17	–	16	–
DYS460	11	–	11	–	11	–
DYS458	15	–	15	–	16	–
DYS19	17	–	15	–	–	–
YGATAH4	–	–	12	–	12	–
DYS448	20	–	20	–	20	–
DYS391	–	–	11	–	–	10
DYS456	16	–	15	–	16	16
DYS390	25	25	24	–	25	25
DYS438	11	–	11	–	–	–
DYS392	11	11	11	–	–	–
DYS518	–	–	40	–	–	–
DYS570	19	–	18	–	19	19
DYS437	14	–	14	–	–	14
DYS385	11,14	–	11,15	–	14	–
DYS449	–	–	33	–	–	–
DYS393	13	–	13	–	13	13
DYS439	10	–	10	–	11	11
DYS481	24	–	23	–	25	–
DYF387S1	36	–	38	–	36	–
DYS533	–	–	12	–	12	–

*DC* dental calculus sample, *T* teeth sample, (–) no amplification obtained possible DNA degradation.

**Table 11:** Summary of results in the Y Filer Plus system—teeth (T) and dental calculus (DC) samples—continued.

Sample	27/T	27/DC	33/T	33/DC	53/T	53/DC
DYS576	–	–	18	18	17	17
DYS389I	13	–	14	–	13	–
DYS635	–	–	–	–	23	–
DYS389II	29	–	–	–	–	–
DYS627	16	–	16	–	–	–
DYS460	11	–	11	–	11	11
DYS458	14	–	15	15	15	15
DYS19	–	–	–	16	18	18
YGATAH4	–	13	13	–	–	–
DYS448	20	–	20	–	20	20
DYS391	–	–	–	–	11	11
DYS456	15	15	17	17	16	16
DYS390	–	–	25	–	26	26
DYS438	11	–	–	–	11	–
DYS392	–	–	11	11	11	–
DYS518	–	–	–	–	42	42
DYS570	19	19	18	18	20	20
DYS437	–	–	14	–	14	14
DYS385	11	11	13,14	14	11	–
DYS449	33	–	–	32	34	–
DYS393	13	13	13	13	13	–
DYS439	10		11	11	11	11
DYS481	–		23	–	23	23
DYF387S1	38		36	36,39	38	–
DYS533	–		–	–	12	–

*DC* dental calculus sample, *T* teeth sample, (–) no amplification obtained possible DNA degradation.

**Table 12:** Summary of results in the Y Filer Plus system—teeth (T) and dental calculus (DC) samples—continued.

Sample	32/T	32/DC	61/T	61/DC	63/T	63/DC
DYS576	17	–	19	19	16	16
DYS389I	13	–	13	–	13	13
DYS635	20	–	24	–	24	24
DYS389II	31	–	31	–	30	30
DYS627	18,19	–	20	–	21	–
DYS460	10	10	10	–	9	9
DYS458	16	16	17	–	16	16
DYS19	17	17	15	–	13	13
YGATAH4	11	11	11	–	11	–
DYS448	20	–	19	–	–	–
DYS391	11	–	–	–	–	10
DYS456	14,15	15	15	15	18	18
DYS390	24	–	24	–	24	24
DYS438	10	–	10	–	10	–
DYS392	11	–	11	–	11	–
DYS518	40	–	39,40	–	–	–
DYS570	20	20	18	–	19	19
DYS437	15	–	15	15	14	–
DYS385	14,15	–	14,15	15	17	–
DYS449	32	32	31	–	31	–
DYS393	13	13	13	13	13	13
DYS439	13	–	13	–	13	–
DYS481	32	–	32	–	21	–
DYF387S1	37,39	–	38	38	35,36	–
DYS533	12	–	12	12	12	–

*DC* dental calculus sample, *T* teeth sample, (–) no amplification obtained possible DNA degradation.

**Table 13:** Summary of results in the Y Filer Plus system—teeth (T) and dental calculus (DC) samples—continued.

Sample	47/T	47/DC	58/T	58/DC	91/T	91/DC
DYS576	17	17	18	18	16	16
DYS389I	13	13	13	–	12	12
DYS635	–	23	–	23	–	–
DYS389II	–	29	32	–	–	–
DYS627	–	16	–	20	19	–
DYS460	11	11	10	10	10	10
DYS458	17	17	17	17	15	15
DYS19	17	17	–	–	–	14
YGATAH4	13	13	–	13	11	11
DYS448	–	–	–	–	20	20
DYS391	–	11	–	–	10	–
DYS456	15	15	15	15	–	14
DYS390	–	–	24	–	23	23
DYS438	–	11	10	–	10	–
DYS392	–	11	–	–	–	–
DYS518	–	40	–	–	–	–
DYS570	19	19	18	18	21	21
DYS437	–	14	15	15	16	-
DYS385	14	11,14	14,15	14,15	13,14	13
DYS449	–	33	31	–	–	–
DYS393	13	13	13	13	13	13
DYS439	10	10	13	13	10	10
DYS481	23	23	–	30	–	25
DYF387S1	–	38	–	–	37	37
DYS533	–	–	–	–	–	–

*DC* dental calculus sample, *T* teeth sample, (–) no amplification obtained possible DNA degradation.

Table 14 summarizes the amplifications obtained in the GlobalFiler and Y-Filer Plus identification system from the test material.

**Table 14:** Summary of calculus and teeth results in the GlobalFiler and Y-Filer Plus systems.

No	Sample	Biological material	GlobalFiler system	YF plus system
1	4	Dental calculus	Degraded profile	Incomplete DNA profile
		Teeth	Incomplete DNA profile	Heavily degraded profile
2	6	Dental calculus	Incomplete DNA profile	Incomplete DNA profile
		Teeth	Incomplete DNA profile	Incomplete DNA profile
3	7	Dental calculus	Degraded profile	Empty profile
		Teeth	Incomplete DNA profile	Incomplete DNA profile
4	11	Dental calculus	Degraded profile	Heavily degraded profile
		Teeth	Incomplete DNA profile	Incomplete DNA profile
5	16	Dental calculus	Heavily degraded profile	Empty profile
		Teeth	Complete DNA profile	Complete DNA profile
6	23	Dental calculus	Incomplete DNA profile	Not obtained
		Teeth	Heavily degraded profile	Not obtained
7	25	Dental calculus	Degraded profile	Heavily degraded profile
		Teeth	Incomplete DNA profile	Incomplete DNA profile
9	27	Dental calculus	Incomplete DNA profile	Heavily degraded profile
		Teeth	Incomplete DNA profile	Incomplete DNA profile
9	30	Dental calculus	Degraded profile	Degraded profile
		Teeth	Complete DNA profile	Complete DNA profile
10	32	Dental calculus	Incomplete DNA profile	Heavily degraded profile
		Teeth	Complete DNA profile	Complete DNA profile
11	33	Dental calculus	Degraded profile	Degraded profile
		Teeth	Incomplete DNA profile	Incomplete DNA profile
12	47	Dental calculus	Complete DNA profile	Incomplete DNA profile
		Teeth	Incomplete DNA profile	Degraded profile
13	53	Dental calculus	Incomplete DNA profile	Degraded profile
		Teeth	Complete DNA profile	Incomplete DNA profile
14	58	Dental calculus	Complete DNA profile	Degraded profile
		Teeth	Incomplete DNA profile	Degraded profile
15	61	Dental calculus	Incomplete DNA profile	Heavily degraded profile
		Teeth	Complete DNA profile	Complete DNA profile
16	63	Dental calculus	Incomplete DNA profile	Degraded profile
		Teeth	Complete DNA profile	Incomplete DNA profile
17	91	Dental calculus	Incomplete DNA profile	Incomplete DNA profile
		Teeth	Complete DNA profile	Incomplete DNA profile

## Discussion

The research aimed to find an alternative source of human DNA with a concentration suitable for amplifying STR and Y-STR markers for identification purposes. This is particularly crucial for remains of historical, museum, or forensic significance, where the nature of the proceedings prohibits disturbing the structure of the skeleton.

In this study has been shown that dental calculus contains human DNA in sufficient quantities for identification purposes. The literature states that it is also a rich reservoir of ancient host biomolecules and human DNA^2^. The initial reports of human DNA content in dental calculus were related to archaeological calculus. Subsequent studies by other authors confirmed the presence of mtDNA and nuclear DNA in calculus samples obtained from archaeological remains. The studies were based on Next-Generation Sequencing (NGS) technology^19,20^.

As mentioned earlier, previous dental calculus studies have mainly focused on obtaining information about dietary habits and the oral microbiome, primarily involving archaeological calculus^7,21^. Other researchers have attempted to determine haplogroup affiliation using mtDNA isolated from dental calculus^22,23^. However, there is a lack of research on the assessment of human DNA content in dental calculus for identification purposes. Analyses of living individuals' dental calculus have also been conducted, which demonstrated the presence of human DNA in this material^11^. By working with forensic DNA analysis kits that are sensitive and allow the use of low-template DNA, we are able to generate a human DNA profile suitable for preliminary identification analyses. The study also shows that the quantity of collected dental calculus is crucial. Samples with a significantly higher amount of calculus (approximately 20 mg) yielded better DNA amplification results. The STR marker analysis kit used in this study is optimized for challenging samples, and dental calculus undoubtedly falls into that category. This kit enabled optimal results despite the degraded samples. When working with highly mineralized materials (bones, teeth, dental calculus), the DNA isolation process is crucial. This type of material is widely recognized as challenging to extract DNA from. Our experience shows that typical lysis reagents are not effective. In this study, the Maxwell® FSC DNA IQ Bone DNA Extraction Kit (Promega) was used for isolation, which proved to be beneficial in this case. The kit includes a demineralization buffer, which is an important element in the initial extraction processes for highly mineralized samples.

Obtaining DNA with good quality and quantity is a crucial aspect of identification studies. The concentration of DNA must be sufficient to proceed with further analysis stages. Forensic investigations often involve challenging samples that are partially degraded, contain low amounts of DNA, and may contain PCR inhibitors. All of these factors affect the time-consuming laboratory processes and the quality of the obtained results^24^. Detecting PCR inhibitors and determining the degradation index (DI) are crucial in the amplification and genotyping process^25^. DNA degradation is visible on electropherograms as the amplification of small fragments that amplify well, and large fragments that are damaged and amplify poorly. In our study, the DNA concentration was determined using the Quantifiler Trio Quantification Kit (ThermoFisher). With this kit, a degradation index ≤ 1 indicates undamaged DNA (the concentration of small and large fragments is approximately equal). Any degradation index > 1 may indicate DNA degradation. Another problem is the presence of PCR inhibitors, which negatively affect amplification. On electropherograms, this appears similar to degradation (large fragments do not amplify). However, inhibition can occur during DNA quantification or amplification stages^26^. Analyzing the total concentration of human DNA from dental calculus obtained in our study, it can be observed that it does not significantly differ from the DNA isolated from teeth. On average, it is approximately 0.04338 ng/µl lower than the average concentration obtained from teeth. These concentrations are practically comparable. It should be emphasized that the total DNA content in human teeth can vary significantly between individuals and even between teeth from the same individual. This depends on the type of teeth sampled, the individual's health status, and chronological age at the time of teeth extraction. These factors influence the proportions of DNA present in the root, pulp, dentin, or crown itself^5,27,28^. In the presented study, we have shown that the degradation index of DNA isolated from dental calculus is much lower than that isolated from teeth. This is consistent with the work of other researchers who have shown that dental calculus is more stable and less contaminated compared to dentin, although it is more fragmented and shorter in length^29^. However, as our study shows, the concentration of DNA in dental calculus is sufficient to obtain a profile suitable for preliminary identification analysis.

Considering the average degradation index of the analysed samples in our study, we can observe that for dental calculus samples, it ranged from 0.47344 to 3.85240, while for teeth samples, it ranged from 1.99240 to 38.15341. It was significantly higher for teeth than for dental calculus, which, in some teeth samples labeled in the GlobalFiler system, affected the quality of amplification. However, upon analysing all the tested samples, it can be concluded that for dental calculus samples, there were samples with an DI > 1, which, according to the manufacturer's specifications^26^, indicates undamaged DNA, as evidenced by the quality of amplification obtained in the GlobalFiler system. In the case of one dental calculus sample (7/DC), the degradation index could not be determined because long fragments did not amplify. However, a degraded DNA profile was obtained from this sample.

The situation is reversed for teeth samples. An DI > 1 could not be achieved for any of the teeth samples. In all samples, it was significantly higher, and in the case of samples 4/T, 6/T, 11/T, and 23/T, it reached high values, indicating significant DNA degradation.

Our study shows that dental calculus contains human DNA useful in forensic analyses. In the event of degradation or fragmentation of human remains or death by fire, teeth and dental calculus may prove more useful in the identification process than other bones. In the case of fire victims, teeth are more protected than other bones due to their morphology and location in the maxilla^5^. Fire victims are people who often have limited opportunities for accurate identification. Our team has extensive experience in identifying fire victims (the Casa plane crash in Poland in Mirosławiec, identification of victims of a nursing home in Kamień Pomorski in Poland, a number of identifications of victims of car accidents where the body is burned, bodies burned as a result of crimes). The first material we reach for is teeth. If the teeth are preserved, the dental calculus will be preserved. The component of teeth is hydroxyapatite, which undergoes recrystallization at high temperatures, which strengthens their structure. The subgingival dental calculus is integrated with the teeth and the human DNA contained in it is embedded in hydroxyapatite. This makes it well preserved. The remaining bones are composed mostly of organic compounds: proteins, mineral salts, calcium carbonate and phosphate, and magnesium. The protein is denatured and calcium oxide is formed from the mineral components of the bones. The age of the victim at the time of death, diet, body weight and diseases are important in the burning process^5,30^.

Dental pulp serves as an excellent source of DNA, but in cases of poor preservation of human remains or lack of consent for interference with the remains, dental calculus proves to be a valuable material. Other researchers have also demonstrated the potential of dental calculus as an investigative tool in forensic science^29^. It is worth considering the process of preparing teeth for isolation as well. Previous genetic analyses conducted by our research group subjected bone material and teeth to standard cleansing procedures, including mechanical and chemical methods^12,18,31,32^. Our study demonstrates that since dental calculus contains human DNA in concentrations comparable to that obtained from teeth dating back to World War II, it is necessary to mechanically clean the teeth, thereby eliminating an additional source of human DNA that could contribute to better amplification of STR markers. In the case of such degraded material as remains from World War II, where extensive decomposition has occurred, every source of DNA useful in the identification process is highly valuable. Further studies are needed to assess the usefulness of dental calculus in the identification process.

Accumulated dental calculus is, on the one hand, a serious oral health problem, a source of periodontal disease, and for forensic geneticists, it is a source of human DNA, as the study proves. The structure and composition of dental calculus make it less susceptible to environmental degradation compared to other hard tissues such as bone and teeth. It is also utilized as a valuable tool in archaeological and microbiological research. Further research on larger numbers of skeletons is needed to fully prove that we can use it to identify a person. Our team often participates in the exhumation of victims from World War II, so we can continue exhuming them.

## Data Availability

The data supporting the conclusions of this study are available from the Ministry of Culture and National Heritage, however, there are limitations to the availability of the data used under license for this study and therefore are not publicly available. However, the data is made available to the authors upon a justified request and with the consent of the Ministry of Heritage and National Culture.
